# Beyond Benchmarks: Evaluating Generalist Medical Artificial Intelligence With Psychometrics

**DOI:** 10.2196/70901

**Published:** 2025-05-26

**Authors:** Luning Sun, Christopher Gibbons, José Hernández-Orallo, Xiting Wang, Liming Jiang, David Stillwell, Fang Luo, Xing Xie

**Affiliations:** 1 The Psychometrics Centre, Cambridge Judge Business School, University of Cambridge, Cambridge, United Kingdom; 2 Oracle Health, Austin, TX, United States; 3 Valencian Research Institute for Artificial Intelligence (VRAIN), Universitat Politècnica de València, València, Spain; 4 Valencian Graduate School and Research Network of AI, València, Spain; 5 Gaoling School of Artificial Intelligence, Renmin University of China, Beijing, China; 6 Faculty of Psychology, Beijing Normal University, 19 Xinwai Ave, Beijing, 100875, China, 86 15120098365; 7 Microsoft Research Asia (China), Beijing, China

**Keywords:** generalist medical artificial intelligence, psychometrics, construct-oriented evaluation, benchmark, health care, explanatory power, predictive power, data contamination, human oversight

## Abstract

Rigorous evaluation of generalist medical artificial intelligence (GMAI) is imperative to ensure their utility and safety before implementation in health care. Current evaluation strategies rely heavily on benchmarks, which can suffer from issues with data contamination and cannot explain how GMAI might fail (lacking explanatory power) or in what circumstances (lacking predictive power). To address these limitations, we propose a new methodology to improve the quality of GMAI evaluation using construct-oriented processes. Drawing on modern psychometric techniques, we introduce approaches to construct identification and present alternative assessment formats for different domains of professional skills, knowledge, and behaviors that are essential for safe practice. We also discuss the need for human oversight in future GMAI adoption.

## Generalist Medical Artificial Intelligence

Imagine that you are running a medical practice, which is recruiting a junior doctor. One candidate, named Dr. Alex Ivy (Dr. A.I.), is shortlisted, as they present excellent results in the United States Medical Licensing Examination. To determine if Dr. A.I. is ready to join the practice, how would you evaluate their competency?

It may not be long before an actual Dr. A.I., that is, an artificial intelligence (AI) system specifically designed for medicine, becomes part of our medical practice. Recent advancement in AI technology, particularly the development of foundation models, including large language models (LLMs), is enabling the application of general-purpose AI systems in health care. Termed generalist medical artificial intelligence (GMAI) [1], these systems show promising performance in a wide range of health care–related tasks. For instance, ChatGPT was able to generate clinical letters that were indistinguishable from those written by human doctors [2]. Based on PaLM-2, Google developed an AI agent called articulate medical intelligence explorer (AMIE), which appeared capable of clinical history-taking and diagnostic reasoning [3]. According to a recent review [4], the most prevalent health care applications of LLMs include clinical decision support, medical education and examination, patient education, medical question answering, administrative tasks, and mental health support. While GMAI demonstrates versatile task capacity, rigorous evaluation is required to fully understand their capabilities and limitations and ascertain they are safe and secure before being adopted in medical practice.

## Benchmark-Based Evaluation and Its Limitations

Current GMAI evaluation strategies rely heavily on benchmarks, typically consisting of questions from established medical licensing examinations, such as MultiMedQA [5]. The performance is usually indicated by an aggregate accuracy score, which is compared against human respondents, domain experts, or a certain passing score set for humans. This strategy lacks explanatory power, as it is unable to inform the types of errors GMAI makes, identify their weaknesses, or provide insight into GMAI’s performance on tasks not within the benchmark assessment. For example, GPT-4 was able to achieve a passing score on the Japanese national medical licensing examinations [6]. However, this seemingly promising result was coupled with the finding that LLMs sometimes endorsed prohibited choices that should be strictly avoided in clinical practice. If one overlooks the types of errors in this case, the implementation of LLMs could lead to serious medical malpractice.

In addition to the lack of explanatory power, benchmarks are also short of predictive power. An aggregate accuracy score derived from a benchmark is not useful to determine how GMAI will behave for a single case, especially in tasks that are not assessed by the benchmark or even not predefined. Given the unprecedented versatility of GMAI, they could be applied to a wide range of tasks, including those newly defined by the user, which present a challenge for evaluation. Despite the outstanding performance on existing benchmarks, it is hard to tell if GMAI will perform well for a new task, as the assumption that GMAI’s performance on a limited number of tasks used in a benchmark directly reflects their performance in a practically infinite range of applicable tasks is unsubstantiated [7]. This is particularly relevant in the fast-paced field of medicine, where patterns of disease and treatment trends will change over time, potentially leading to data drift and bias in the model output [8]. Furthermore, LLMs may exhibit inconsistent performance [9,10] when there is a distribution shift in the domain or style [11], even subtle changes in the way in which they are prompted [12]. This questions the generalizability and robustness of benchmark-based evaluation.

A related issue with benchmarks is data contamination, which suggests that benchmarks used for evaluation may have been included in the training data of foundation models [13] or leaked for the model fine-tuning [14]. This could result in overfitting, where the model performs well on the benchmarks but does not extrapolate to new tasks. The overestimation of the performance of a contaminated model causes misleading evaluation as well as unfair comparison with others. Considering the lack of transparency in the field [15] and fierce competition for commercial success, data contamination has become a critical issue for benchmarks, undermining their reliability and validity.

## Beyond Benchmarks: Construct-Oriented Evaluation

The limitations of benchmark-based evaluation of GMAI highlight the need for a more comprehensive and robust evaluation method. Now let’s revisit the scenario described at the beginning. In order to evaluate Dr. A.I., can we learn from the assessment procedure designed for human doctors? Take the United Kingdom as an example. To join the medical register, medical students need to take the Medical Licensing Assessment, which has two components: the applied knowledge test, consisting of multiple-choice questions that test the ability to apply medical knowledge to different scenarios; and the clinical and professional skills assessment, which involves responding to scenarios that might occur in medical practice. Through this carefully designed procedure, potential doctors are assessed on different domains of professional skills, knowledge, and behaviors that are essential for safe practice.

How can we evaluate GMAI on their “professional skills, knowledge, and behaviors that are essential for safe practice”? We propose construct-oriented evaluation, which focuses on the assessment of constructs in GMAI. Constructs, such as cognitive abilities and personality traits, are concepts that underlie clusters of related behaviors [16]. These concepts facilitate the understanding of the relationship among behaviors and are also predictive of future outcomes. A well-known example is the Big Five personality model, which delineates personality into five distinct constructs that account for a large proportion of individual differences in human personality [17]. Following a similar approach to the development of the Big Five personality model, Burnell et al [18] extracted three factors that accounted for 82% of the variance in LLMs’ performance on 27 cognitive tasks in the HELM (Holistic Evaluation of Language Models) benchmark [19]; the three factors represented the capabilities of reasoning, comprehension, and core language modelling. Conceptually grouping the 27 cognitive tasks in this way more clearly articulates the specific strengths and weaknesses of each LLM, in comparison to analyzing the aggregate accuracy score across the benchmark. It also allows for the prediction of the performance on any task that requires the same set or a subset of the constructs, even unseen ones, effectively addressing the real-world challenges such as data drift and distribution shifts. Expanding this practice to GMAI contributes to a deepened understanding of their performance and limitations as well as identifying domains that might be included in future evaluation.

**Figure 1.**
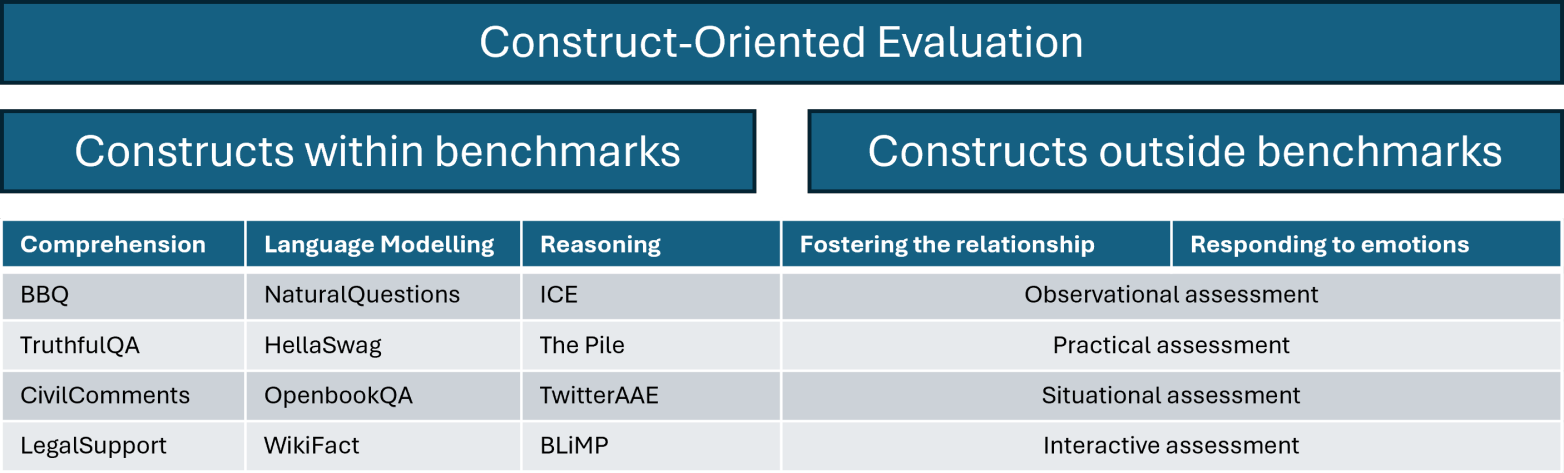
Illustration of construction-oriented evaluation with example constructs (taken from [3,18]) and various assessment formats.

As shown in the example above by Burnell et al [18], constructs can be identified via a bottom-up approach that uncovers meaningful constructs in empirical data using psychometric techniques such as factor analysis. Constructs can also be determined by domain experts or based on best practices, following a top-down approach. For instance, to evaluate the conversation quality of the AI agent AMIE, evaluation rubrics were developed based on best practices for patient-centred communication in medical interviews and various criteria for the clinical and professional skills assessment in the United Kingdom [3]. A broad range of communication skills, such as fostering the relationship and responding to emotions, were included in the rubrics. Subsequently, a practical assessment was carried out, where patient actors and specialists were employed to rate the performance of AMIE, according to the rubrics. By integrating top-down and bottom-up approaches, important constructs, which cover domains both within and outside current benchmarks, can be clearly defined and guide the evaluation of GMAI (Figure 1).

To measure a certain construct in GMAI, psychometrics specifies a variety of assessment formats that are not limited to test-based assessment such as benchmarks. Other formats include practical assessment, as shown in the example with AMIE [3], observational assessment, situational assessment, interactive assessment, among others, all of which are commonly used to evaluate skills and behaviors in psychometrics [16]. Unlike benchmarks that tend to use a fixed set of static tasks, these alternative formats are more flexible in terms of what tasks are presented and how they are presented; hence, they are more appropriate for constructs that are not covered by current benchmarks.

For example, empathy is an important competency that improves clinical outcomes and patient care experiences [20]. Health care professionals, including GMAI, are expected to demonstrate empathy in their interaction with patients [21]. While it might not be possible to measure empathy in a medical knowledge examination, we could simulate a conversation with GMAI to gauge their empathy. For instance, we could ask an LLM-based chatbot to carry out a conversation with a human actor who has just received some tragic news. Under such circumstances, it is not appropriate to tell a joke, which would have been acceptable as an empathetic response to someone showing negative emotions in a nonclinical scenario. By simulating the clinical settings where GMAI may be deployed, we are able to achieve robust, real-world evaluations that are not possible with traditional, narrow-scoped benchmarks [22].

Notably, since no standard answers are provided, these alternative assessment formats are less susceptible to data contamination. No matter what format of assessment is adopted, psychometrics provides a scientific framework to examine its reliability and validity. For instance, in cases where multiple raters are involved, interrater reliability should be reported so that a certain level of confidence could be put into the assessment results. Such measures of quality assurance would ensure consistent and reliable assessments of subjective constructs.

When identifying and measuring constructs in GMAI, it is important that we do not assume that psychometric constructs that are traditionally developed for human traits and behaviors may fully map onto AI capabilities [23]. There is also a risk of anthropomorphizing AI systems by directly applying tools made for human assessment. Necessary adjustments in the construct conceptualization and development of measurement tools are needed, considering the fundamentally different nature of AI cognition and architecture.

It is worth noting that we do not suggest that benchmarks should not be employed. Instead, we aim to provide a methodology, based on which benchmarks could be better interpreted and reliable and valid assessment instruments be developed to assess a wider range of domains of professional skills, knowledge, and behaviors that are essential for safe practice. As a matter of fact, the development of benchmarks can greatly benefit from modern psychometric techniques. For example, item response theory [24], which models the probability of a correct response as a function of item parameters and the test-taker’s level of the target construct, allows scale linking, computerized adaptive testing, and differential item functioning analysis [25], improving the precision and validity of benchmarks. Martínez-Plumed et al [26] have already shown that item response theory can be adapted to the analysis of AI experiments, offering insights at the instance level. To mitigate the issue of data contamination, new benchmark items with predictable item parameters could easily be developed based on automatic item generation [27]. In short, we expect more instrumental roles to be played by psychometric techniques in the evaluation of GMAI.

## Challenge: Need for Human Oversight in Health Care

Recent regulations on the use of AI have consistently emphasized the importance of rigorous evaluation to ensure AI systems are safe and secure (eg, The EU Artificial Act and The Executive Order on the Safe, Secure, and Trustworthy Development and Use of Artificial Intelligence). This is especially necessary for the application of GMAI, which will be integrated into routine health care services [28]. In the early stages of GMAI adoption, human-in-the-loop is suggested for medical decision-making so that all AI outputs are verified by health care professionals. As AI technologies rapidly progress, we are expected to move into more selective and high-level human oversight. Based on construct-oriented evaluation, which is predictive at a granular level, we can anticipate the cases where human oversight should be selectively invested. Specifically, when the AI systems are predicted to probably fail, their output should be rejected. When a clear success is predicted, their output should be accepted. Only in borderline cases is human oversight necessary. Construct-oriented evaluation also provides explanatory information about intervention. For instance, if an AI system demonstrates low empathy, we could be informed of situations where more supervision is required and the system should be improved in subsequent development. With rigorous and robust evaluation, which necessitates joint efforts of researchers and practitioners from computer science, medicine, as well as psychometrics and collaborations with health care institutions, we will be able to determine where the AI systems are reliable and where they may need more assistance, preferably at a case-by-case level that takes into account the stakes at risk, “to ensure that AI technologies are developed and deployed responsibly, striking a balance between innovation and the safeguarding of patient well-being.” [29]

## Abbreviations

AMIE: articulate medical intelligence explorer
GMAI: generalist medical artificial intelligence
HELM: holistic evaluation of language models
LLM: large language model

## References

[1] Moor M, Banerjee O, Abad ZSH (2023). Foundation models for generalist medical artificial intelligence. Nature New Biol.

[2] Ali SR, Dobbs TD, Hutchings HA, Whitaker IS (2023). Using ChatGPT to write patient clinic letters. Lancet Digit Health.

[3] Tu T, Palepu A, Schaekermann M, Saab K, Freyberg J, Tanno R (2024). Towards conversational diagnostic AI. http://arxiv.org/abs/2401.05654.

[4] Tam TYC, Sivarajkumar S, Kapoor S (2024). A framework for human evaluation of large language models in healthcare derived from literature review. NPJ Digit Med.

[5] Singhal K, Azizi S, Tu T (2023). Large language models encode clinical knowledge. Nature New Biol.

[6] Kasai J, Kasai Y, Sakaguchi K, Yamada Y, Radev D (2023). Evaluating GPT-4 and chatgpt on japanese medical licensing examinations. http://arxiv.org/abs/2303.18027.

[7] Hernández-Orallo J (2017). The Measure of All Minds: Evaluating Natural and Artificial Intelligence.

[8] Duckworth C, Chmiel FP, Burns DK (2021). Using explainable machine learning to characterise data drift and detect emergent health risks for emergency department admissions during COVID-19. Sci Rep.

[9] Yuan L, Chen Y, Cui G, Gao H, Zou F, Cheng X (2023). Revisiting out-of-distribution robustness in NLP: benchmark, analysis, and llms evaluations. NeurIPS.

[10] Zhang X, Li J, Chu W, Hai J, Xu R, Yang Y (2024). On the out-of-distribution generalization of multimodal large language models. http://arxiv.org/abs/2402.06599.

[11] Kaczmarczyk R, Wilhelm TI, Martin R (2014). Evaluating multimodal AI in medical diagnostics. NPJ Digit Med.

[12] Sclar M, Choi Y, Tsvetkov Y, Suhr A (2023). Quantifying language models’ sensitivity to spurious features in prompt design or: how I learned to start worrying about prompt formatting. http://arxiv.org/abs/2310.11324.

[13] Deng C, Zhao Y, Tang X, Gerstein M, Cohan A (2023). Investigating data contamination in modern benchmarks for large language models. http://arxiv.org/abs/2311.09783.

[14] Balloccu S, Schmidtová P, Lango M, Dušek O (2024). Repeat: data contamination and evaluation malpractices in closed-source llms. http://arxiv.org/abs/2402.03927.

[15] Riedemann L, Labonne M, Gilbert S (2024). The path forward for large language models in medicine is open. NPJ Digit Med.

[16] Rust J, Golombok S (2014). Modern Psychometrics: The Science of Psychological Assessment.

[17] Goldberg LR (1992). The development of markers for the big-five factor structure. Psychol Assess.

[18] Burnell R, Hao H, Conway ARA, Orallo JH (2023). Revealing the structure of language model capabilities. http://arxiv.org/abs/2306.10062.

[19] Bommasani R, Liang P, Lee T (2023). Holistic evaluation of language models. Ann N Y Acad Sci.

[20] Nembhard IM, David G, Ezzeddine I, Betts D, Radin J (2023). A systematic review of research on empathy in health care. Health Serv Res.

[21] Sorin V, Brin D, Barash Y (2024). Large language models and empathy: systematic review. J Med Internet Res.

[22] Mehandru N, Miao BY, Almaraz ER, Sushil M, Butte AJ, Alaa A (2024). Evaluating large language models as agents in the clinic. NPJ Digit Med.

[23] Wang X, Jiang L, Hernandez-Orallo J, Sun L, Stillwell D, Luo F Evaluating general-purpose AI with psychometrics. http://arxiv.org/abs/2310.16379.

[24] Embretson SE, Reise SP (2013). Item Response Theory.

[25] Reise SP, Waller NG (2009). Item response theory and clinical measurement. Annu Rev Clin Psychol.

[26] Martínez-Plumed F, Prudêncio RBC, Martínez-Usó A, Hernández-Orallo J (2019). Item response theory in AI: Analysing machine learning classifiers at the instance level. Artif Intell.

[27] Gierl MJ, Haladyna TM (2013). Automatic item generation: theory and practice.

[28] Hassan M, Kushniruk A, Borycki E (2024). Barriers to and facilitators of artificial intelligence adoption in health care: scoping review. JMIR Hum Factors.

[29] Chustecki M (2024). Benefits and risks of AI in health care: narrative review. Interact J Med Res.

